# Five-Year Outcomes of Patients with Pompe Disease Identified by the Pennsylvania Newborn Screen

**DOI:** 10.3390/ijns11010016

**Published:** 2025-02-24

**Authors:** Hayley A. Ron, Owen Kane, Rose Guo, Caitlin Menello, Nicole Engelhardt, Shaney Pressley, Brenda DiBoscio, Madeline Steffensen, Sanmati Cuddapah, Kim Ng, Can Ficicioglu, Rebecca C. Ahrens-Nicklas

**Affiliations:** 1 Hackensack University Medical Center at Hackensack Meridian Health, Hackensack, NJ 07601, USA; 2 Department of Pediatrics, Perelman School of Medicine, University of Pennsylvania, Philadelphia, PA 19104, USA; 3 Cone Health, Greensboro, NC 27401, USA; 4 Section of Biochemical Genetics, Division of Genetics, Children’s Hospital of Philadelphia, Philadelphia, PA 19104, USA

**Keywords:** Pompe disease, late-onset Pompe disease, infantile-onset Pompe disease, newborn screen, enzyme replacement therapy, alglucosidase alfa, alpha-glucosidase (GAA), glucotetrasaccharides (Hex4)

## Abstract

Pennsylvania started newborn screening for Pompe disease (PD) in 2016. As a result, the prevalence of PD has increased with early detection, primarily of late-onset Pompe disease (LOPD). No clear guidelines exist regarding if and when to initiate enzyme replacement therapy (ERT) in patients identified through a newborn screen (NBS). To help define the natural history and indications for starting ERT, we present the long-term follow-up data of 45 patients identified through NBS from 2016 to 2021. These patients were evaluated at regular intervals through our multi-disciplinary clinic at the Children’s Hospital of Philadelphia (CHOP) with physical examinations, physical therapy evaluations, muscle biomarkers including creatine kinase (CK), aspartate aminotransferase (AST), alanine aminotransferase (ALT), and hexosaminidase 4 levels (Hex4), as well as cardiac evaluation at certain points in time. We found that newborn screening of acid alpha-glucosidase (GAA) enzyme detected primarily LOPD. One case of infantile-onset PD (IOPD) was detected. Muscle biomarkers in LOPD were elevated at birth and showed a general downward trend over time. NBS GAA levels and initial CK levels helped to differentiate LOPD cases from unaffected infants (carriers, pseudodeficiency alleles), while Hex4 was not a meaningful discriminator. On repeat NBS, there was a significant difference between mean GAA levels for the unaffected vs. compound heterozygote groups and unaffected vs. homozygote groups for the common splice site pathogenic variant (c.-32-13T>G). Echocardiogram and electrocardiogram (EKG) are essentially normal at the first evaluation in LOPD. One LOPD patient was started on ERT at age 4.5 months. Continued data collection on these patients is critical for developing management guidelines, including timing of ERT and improved genotype–phenotype correlation.

## 1. Introduction

Pompe disease (glycogen storage disease type II) is a disorder of glycogen metabolism caused by deficiency of the lysosomal enzyme acid alpha-glucosidase (GAA). Deficiency of this enzyme prevents the normal degradation of glycogen, leading to its accumulation in the lysosomes of all tissues. Skeletal, cardiac, and smooth muscle tissue are most notably affected, leading to a spectrum of clinical findings. Pompe disease (PD) is inherited in an autosomal recessive manner, caused by pathogenic variants of the GAA gene located on chromosome 17q25.2-q25.3 [1]. There is a more severe infantile-onset form and a later-onset form with variable severity.

Classic infantile-onset Pompe disease (IOPD) presents in the first months of life. They present in the neonatal period with hypertrophic cardiomyopathy and generalized muscle weakness. Clinically, this manifests with feeding difficulties, failure to thrive, respiratory complications, and/or delayed motor milestones. If untreated, these patients usually die within the first year of life. Physical exam findings may include macroglossia, hepatomegaly, and generalized hypotonia or floppiness.

Patients with late-onset Pompe disease (LOPD) can have broad phenotypic variation. Due to residual enzyme activity, these individuals are less severely affected than those with IOPD. Individuals with LOPD can present anytime from early childhood to adulthood with the predominant finding of slowly progressing muscle weakness [2]. Other complaints may include gait abnormalities, respiratory insufficiency, and fatigue. Notably, they lack the cardiac involvement that is so prominent in IOPD. Because LOPD is rare with nonspecific symptoms, many patients encounter a significant diagnostic delay. The mean diagnostic delay has been reported to be as long as 13 years in one Belgian study [3] and 7.4 years in an Austrian study [4].

The severity of findings typically correlates inversely with the residual enzyme levels [5]. In classic IOPD, infants have less than 1% residual enzyme activity. In children and adults with LOPD, the level of residual enzyme is higher but typically less than 30% [6]. Carriers have about 50% of the average normal enzyme activity [6]; to our knowledge, there are no reports of affected carriers.

Enzyme replacement therapy (ERT) with alglucosidase alfa (Myozyme^®^, Genzyme^®^) was approved for patients with all forms of Pompe disease in 2006 in the United States and the European Union. Open-label studies of IOPD showed that treatment with ERT prolonged survival and improved respiratory status in patients who started therapy before the age of 6 months [7]. In 2021, avalglucosidase alfa-ngpt (Nexviazyme^®^) was approved by the FDA for the treatment of LOPD in patients one year of age and older. In 2023, cipaglucosidase alfa-atga (POMBILITI^®^) in combination with miglustat (Opfolda^®^) was approved for treatment of adults with LOPD who are not improving on their current ERT regimen. Patients who receive ERT show marked improvement in cardiomyopathy and gains in motor and functional skills.

No clear guidelines exist regarding when to initiate ERT in LOPD patients identified through NBS. Several studies have evaluated the efficacy and safety of ERT in LOPD from 3 years of age and older; however, these patients were not incidentally identified through newborn screening. In general, walking distance was most improved by ERT [8,9], respiratory function was improved or stabilized [9,10], and muscle strength showed the least significant improvement [8,11].

Data collection for LOPD identified through newborn screening will help determine the genotype–phenotype correlation and indications for starting ERT. In a large prospective study in Taiwan, newborn screening identified 39 cases of LOPD and 16 cases of IOPD between 2005 and 2018 [12]. The most common variant in their LOPD cohort was c.[752C>T;761C>T]. Eight of the thirty-nine cases with LOPD were treated with ERT. The indications for therapy included persistent CK elevation, elevated CK with cardiomyopathy, and hypotonia with delayed gross motor development. Motor performance improved in all patients following ERT. CK and Hex4 were improved in all subjects but were not sustained for some. The correlation of genotype with initial biochemical profile was inconclusive, but 41% of LOPD subjects had an initial CK > 200 U/L. All IOPD subjects had an initial CK > 300 U/L [12].

To help define the natural history of LOPD identified through NBS and to advance our understanding of when to start ERT, we present the long-term follow-up data of 45 patients identified through the Pennsylvania NBS program from 2016 to 2021. The subjects born between February 2016 and December 2019 were initially described by Ficicioglu et al. in 2020 [2]. In their study, 31 newborns were diagnosed with LOPD, and two were diagnosed with IOPD. Thirty patients were classified as “suspected LOPD” due to compound heterozygous pathogenic variant and VUS, or two VUS. The remainder were classified as having pseudodeficiency (*n* = 15), carriers (*n* = 35), or false positive (*n* = 2). Twelve patients were homozygous for the common splice site mutation (c.-32-13T>G), and eleven other cases were compound heterozygotes that carried the splice site mutation. The median CK and ALT values (but not Hex4 or AST) were significantly higher in LOPD subjects compared with suspected LOPD subjects and carriers/pseudodeficiency. There was not a statistically significant difference in CK levels between the homozygotes and other genotypes.

The present study provides longitudinal follow-up data from this cohort and includes newly identified cases diagnosed and treated at CHOP.

## 2. Materials and Methods

### 2.1. Newborn Screening Follow-Up Protocol

Pompe disease was added to the recommended universal screening panel (RUSP) in February 2015. Pennsylvania started screening in February 2016. Blood for the newborn screen is obtained from the infant’s heel between 24 and 48 h of life and applied to filter paper. In Pennsylvania, the dried blood spot is sent to the PerkinElmer newborn screening laboratory located in Pittsburgh, PA. GAA enzyme activity is measured in a CLIA-certified clinical diagnostic lab via Flow Injection Tandem Mass Spectrometry (FIA/MS/MS). If the enzyme is below the cutoff value of <2.10 mol/L/h on the first screen (herein referred to as NBS1), a second sample is requested. If the level on the second newborn screen (herein referred to as NBS2) is deficient, *GAA* sequencing analysis is performed using next-generation sequencing (NGS). Copy number variation in three exons or more is reported.

Newborns with low enzyme activity and at least one variant (pathogenic, likely pathogenic, or variant of unknown significance) are referred to a metabolic center. Newborns with low enzyme activity and a pseudodeficiency allele do not require referral to a metabolic center but can be referred by pediatricians for genetic counseling at the pediatrician’s discretion.

At the initial visit, the patient is classified as IOPD, LOPD, unknown onset, or carrier based on genotype and confirmatory enzyme activity. Unknown-onset patients carry one pathogenic variant and one or two VUS. Carriers, patients with pseudodeficiency alleles only, or no identified variants do not require further follow-up.

Confirmed IOPD cases undergo appropriate immune modulation therapy and initiation of ERT. All LOPD or unknown-onset cases undergo regular clinical, psychomotor, and biochemical screening to monitor for early symptoms of disease.

### 2.2. Longitudinal Follow-Up Protocol

Regular screening laboratory tests include aspartate aminotransferase (AST), alanine aminotransferase (ALT), creatine kinase (CK), and urine glucose tetrasaccharide (Glc4 or Hex4) at 4–6-month intervals for the duration of the study period. Regular EKG and echocardiogram evaluations are initiated in infancy. If normal, cardiac surveillance occurs at 6-month intervals in the first year and is spaced to annual visits thereafter.

Patients are evaluated by a metabolic geneticist at least every 4–6 months. At each follow-up visit, a physical exam is conducted with an emphasis on cardiovascular and musculoskeletal systems. Anthropometric evaluations are recorded at each visit (height, weight, and head circumference), and growth trends are followed. Physical therapy evaluations are conducted using the Alberta Infant Motor Scale (AIMS) from age 0 to 18 months and the Peabody Developmental Motor Scale from 12 to 18 months until age 5. Laboratory evaluation includes CK, AST, ALT, and Hex4.

### 2.3. Statistical Analysis

All statistical analyses were performed using GraphPad Prism version 9.0 for Mac GraphPad Software, Boston, MA, USA, www.graphpad.com. Figures were prepared in GraphPad Prism. Analysis was done using one-way analysis of variance (ANOVA) followed by Tukey’s multiple comparison test to compare means between genotype groups and onset groups. Third-order polynomial interpolation was used to evaluate the trend of biochemical values over time.

## 3. Results

Between February 2016 and January 2021, a total of 45 newborns who screened positive were enrolled in the present study. The final diagnosis in twenty subjects was LOPD, in one subject was IOPD, in 10 subjects was unknown onset. There were three pseudodeficiency cases, one subject with a normal genotype, and nine carriers (summarized in Table 1). One case was found to have one pseudodeficiency allele and was not seen for evaluation.

### 3.1. Muscle Biomarkers Downtrend over Time in LOPD

**Figure 1:**
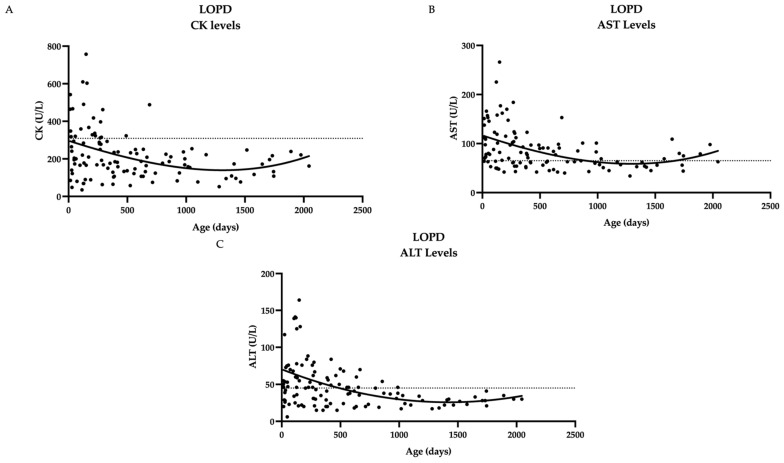
Biochemical markers over time in twenty LOPD patients show a general downward trend over time. (**A**) CK levels over time (**B**) AST levels over time (**C**) ALT levels over time. Dashed line represents upper limit of normal, which is 305 U/L for CK, 65 U/L for AST, 45 U/L for ALT; not included for Hex4 since norms vary by age. Curve fitting with a third-order polynomial is shown.

Serial measurements of biomarkers in patients with LOPD were collected. Several patients had initial elevations that decreased over time (CK, AST, ALT) (Figure 1).

**Figure 2:**
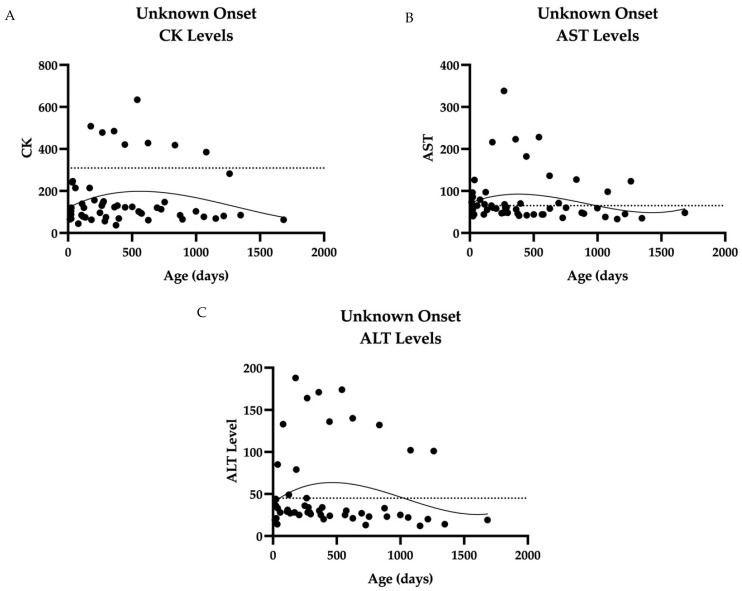
Biochemical markers over time in ten unknown-onset cases: (**A**) CK levels over time, the majority of which are <200 with one outlier, subject 28. (**B**) AST levels over time. Most levels cluster between 40 and 100 U/L, with one outlier, CHOP28. (**C**) ALT levels over time. The majority of ALT levels measured below 50 U/L, with two outliers. Data were collected from 15 days to 696 days of age in ten patients. Dashed line represents upper limit of normal, which is 309 U/L for CK, 65 U/L for AST, and 45 U/L for ALT. Curve fitting with a third-order polynomial is shown.

Muscle biomarkers also show a downtrend over time in Unknown-onset PD. The linear regression of biochemical values over time (CK, AST, ALT) shows a downward trend (Figure 2). Several patients experienced a slight increase in levels before decline. Hex4 reference ranges vary by age group, and the detected levels were within the normal range in this cohort.

**Figure 3:**
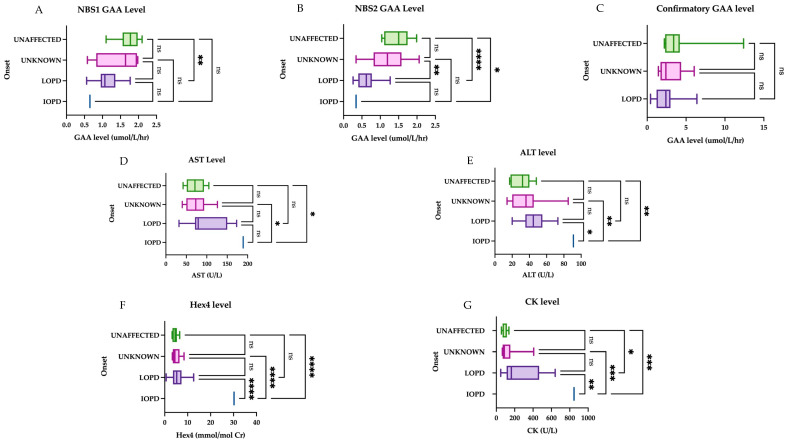
Initial muscle biomarker levels by onset. The first group represents the unaffected group (normal genotype, carriers, and peseudodeficiency alleles), followed by unknown onset, late onset, and infantile onset: (**A**) There is a statistically significant difference between the means for the unaffected group and the LOPD group on NBS1. (**B**) The unaffected and LOPD groups were most significantly different, followed by LOPD vs. unknown onset, and finally IOPD vs. unaffected. (**C**) The comparison of means showed no significant difference in initial GAA level between groups. (**D**) AST was only a useful comparator against the IOPD case; comparison of means between other groups was not significant. (**E**) ALT was only a useful comparator against the IOPD case; comparison of means between other groups was not significant. (**F**) Comparison of means between Hex4 and other groups was statistically significant when comparison involved the IOPD case. (**G**) For CK levels, the most significant difference is seen between unaffected vs. IOPD and unknown vs. IOPD, followed by LOPD vs. IOPD. There was also a statistically significant comparison of means between the unaffected vs. LOPD, but less so. Groups were compared with one-way ANOVA followed by Tukey’s multiple comparison tests. * = *p* < 0.05, ** = *p* < 0.01, *** = *p* < 0.001, **** = *p* < 0.0001, ns = nonsignificant.

Initial GAA and CK levels help to differentiate LOPD cases from unaffected infants, while Hex4 was not a meaningful discriminator. When comparing biomarkers between groups (Figure 3), there was a statistically significant difference between the late-onset group and the unaffected group (including normal genotype, carriers and pseudodeficiency) for NBS1 level (mean 1.129 and 1.733 µmol/L/h, respectively, *p*-value of 0.0029), NBS2 level (mean 0.6274 and 1.478 micromole/L/h, respectively, *p*-value <0.0001), and confirmatory CK level (mean 268 and 97 U/L, respectively, *p*-value <0.001). The comparison of mean Hex4 levels was statistically significant only in comparison with the IOPD group, i.e., IOPD vs. LOPD, IOPD vs. unknown, and IOPD vs. unaffected, with a *p*-value of <0.0001 for all three groups.

**Figure 4:**
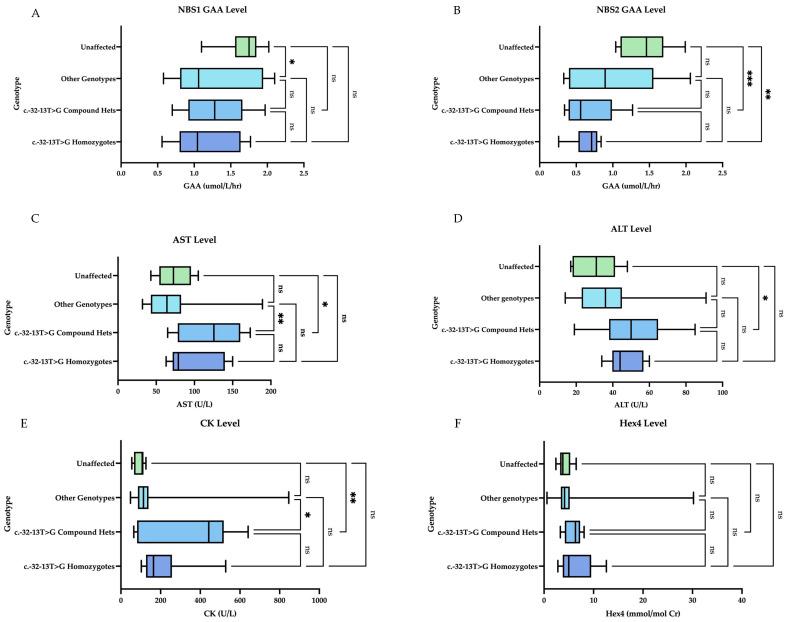
Initial muscle biomarker levels by genotype. Homozygotes indicates homozygosity for the common splice site variant (c.-32-13T>G). Compound hets indicate heterozygosity for the common splice site variant (c.-32-13T>G). Other genotypes represent various combinations of all other VUS and pathogenic variants. Unaffected represents normal genotypes, carriers, pseudodeficiency: (**A**) On NBS1, there is a statistically significant comparison of means between the unaffected group and other genotypes. (**B**) On NBS2, there is a statistically significant comparison of means for unaffected vs. compound hets and unaffected vs. homozygotes. (**C**) For AST level, there is a statistically significant comparison of means for other vs. compound hets and unaffected vs. compound hets. (**D**) For ALT level, the only statistically significant comparison of means was between unaffected vs. compound hets. (**E**) For CK levels, there was a statistically significant comparison of means between the unaffected and compound hets group and the other genotypes vs. compound hets. (**F**) For Hex4 level, there was no statistically significant comparison of means between the groups. Groups were compared with one-way ANOVA followed by Tukey’s multiple comparison tests. * = *p* < 0.05, ** = *p* < 0.01, *** = *p* < 0.001, ns = nonsignificant.

Muscle biomarkers are elevated in LOPD patients heterozygous or homozygous for the common splice site variant (c.-32-13T>G). Hex4 does not vary significantly by genotype (Figure 4). When comparing genotype groups, we found a statistically significant difference between unaffected infants (including carrier and pseudodeficiency) and compound heterozygotes (heterozygous for the common splice site mutation (c.-32-13T>G) for NBS2 levels (mean 1.43 and 0.68, respectively, *p*-value 0.001), initial CK levels (mean 93 and 367 U/L, respectively, *p*-value 0.0043), initial AST levels (74 U/L and 123 U/L, respectively, *p*-value 0.0199), and ALT levels (mean 31 and 51 U/L, respectively, *p*-value 0.0396). There was also a statistically significant difference between the unaffected and homozygous patients for NBS2 levels (mean 1.43 and 0.65 µmol/L/h, respectively, *p*-value 0.004). There was no statistically significant difference among the mean Hex4 levels by genotype.

### 3.2. Cardiac

#### 3.2.1. Electrocardiogram (EKG) Is Normal in LOPD

EKG findings were available for 28 patients at their initial evaluation (1—infantile, 17—late onset, 7—unknown, 2—carriers, 1—pseudodeficiency). In the subject with IOPD, EKG showed diffuse T-wave inversions and biventricular hypertrophy with strain. Most of the remaining EKGs (22/27) are normal. Abnormal findings in the late-onset population included right axis deviation (2/17), hypertrophy (1/17), and borderline QTc interval (1/17). In the unknown-onset group, all EKGs are normal except one that showed biventricular hypertrophy. There are two carriers and one pseudodeficiency with normal EKGs.

#### 3.2.2. Echocardiograms Are Essentially Normal at Initial Evaluation in LOPD Infants

Echocardiogram findings were available for 30 patients at their initial evaluation (1—infantile, 17—late onset, 9—unknown, 2—carriers, 1—pseudodeficiency). Twelve out of thirty had no remarkable findings. PFO was the most common finding identified in 14/30 subjects. Hypertrophy (with consistent EKG findings) and qualitatively subtle hypertrophy of LV apex with PFO were identified in two different late-onset patients. Dilatation and hypertrophy of RV were found in one unknown-onset patient. The infantile-onset patient had hypertrophy. No clinically significant cardiac disease developed in any of the LOPD or unknown-onset patients over the course of the study. As our understanding of this patient population evolved and universally normal results were seen in early practice, the frequency of cardiac evaluations decreased over time. The remainder of the findings are summarized in Table 1.

#### 3.2.3. Clinical Case of Initiation of ERT for a Patient with Late-Onset PD

**Figure 5:**
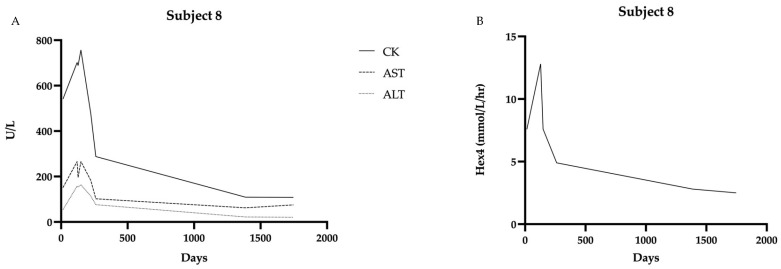
Biochemical markers over time in Subject 8 showing decline in all markers after starting ERT at 4.5 months of age: (**A**) CK, AST, and ALT levels over time. (**B**) Hex4 levels over time. Data were collected from 15 days to 1745 days of age.

One individual in the cohort (Subject #8) with a diagnosis of presumed late-onset Pompe disease was started on ERT at age 4.5 months. On newborn screening, the first GAA level was 0.7 µmol/L/h, and the second level was 0.34 µmol/L/h. The genotype was heterozygous for the common intronic variant (c.-32-13T>G) and a pathogenic variant, c.2560 C>T (p.Arg854*). The newborn presented to our clinic for initial evaluation and confirmatory testing at 15 days of age. Initial CK was 542 U/L (upper limit of normal is 305 U/L), AST 152 U/L (65 U/L) and ALT 55 U/L (45 U/L). Hex4 was 7.6 mmol/mol Cr (<19 mmol/Cr). EKG was unremarkable; echocardiogram identified possible subtle hypertrophy of LV apex. CK, AST, and ALT remained elevated well above other individuals in the late-onset cohort (Figure 5). Motor development, as measured by the AIMS, was at the 25th percentile at 3 months of age and fell to the 10th percentile by 4.5 months of age. Given the motor findings in conjunction with the biochemical markers, the decision was made to initiate ERT by 5 months of age. After initiating therapy, her CK normalized within 3 months, and her AIMS percentage increased to the 75th percentile by 8.5 months of age. Cardiac surveillance has revealed continued normal structure and function.

## 4. Discussion

In the present study of PD identified through Pennsylvania newborn screening, both IOPD and LOPD were detected. We describe the cardiac and biochemical findings at initial presentation and those collected over time in our multidisciplinary clinic. We describe the rationale for initiating ERT at 4.5 months of age in a single late-onset case.

In Pennsylvania, full gene sequencing is performed on the repeat newborn screen specimen if enzyme activity is again below the cutoff. Having the genetic result for the initial consultation helps to more efficiently classify the onset and form a management plan. This decreases some of the anxiety and uncertainty surrounding the initial NBS result. Genetic analysis identifies pseudodeficiency alleles, for which consultation at a metabolic center and follow-up studies are not necessary. Including genetic analysis with the NBS also equalizes access to genetic testing, which otherwise has variable insurance coverage.

The leaky GAA splice site variant c.-32-13T>G was found in at least one allele in 19 subjects (42%). Of those subjects, eight are homozygotes, ten are compound heterozygotes, and one is a carrier. Homozygotes for this variant are described as manifesting the full clinical spectrum of LOPD and have overlapping phenotypic findings with the heterozygotes [13]. Clinical features include adult-onset disease with proximal leg weakness, CK elevations, and reduced GAA activity [13]. This variant is never implicated in IOPD.

We attempted to correlate genotype with NBS results (enzyme level) and muscle biomarkers. In the present cohort, the NBS2 level and the confirmatory CK, AST, and ALT levels were significantly different between the unaffected patients (carriers, pseudodeficiency alleles, normal genotype) and the compound heterozygotes for the c.-32-13T>G variant. There was also a significant difference in NBS2 values when comparing unaffected patients and homozygotes. There was not a significant difference between the compound heterozygotes and homozygotes for any of the biomarkers. Additionally, there was not a statistically significant difference in initial Hex4 levels between the genotypes (excluding the patient with IOPD).

We also attempted to correlate the final diagnosis (IOPD vs. LOPD vs. unknown) with enzyme levels and muscle biomarkers. The classification of IOPD, LOPD, unknown onset, or carrier is based on genotype and confirmatory enzyme levels. NBS2 and confirmatory CK levels were significantly different between the unaffected patients and those classified as LOPD. The unknown-onset and LOPD cases also had significantly different NBS2 levels. Hex4 level was only helpful in differentiating IOPD from all other classifications (unknown, LOPD, unaffected).

Cardiac findings were normal in the majority of LOPD and unknown-onset cases at the time of diagnosis or had incidental findings such as PFO. One EKG was suggestive of hypertrophy in the LOPD group, but the echocardiogram did not confirm this finding. Cardiac findings are expected to be normal in neonates with LOPD, as demonstrated by other newborn screen cohorts [12,14]. This was corroborated in our cohort, such that no clinically significant cardiac disease was identified in LOPD or unknown-onset patients during the study period.

Taken together, the above findings suggest that NBS2 and CK levels seem to be more meaningful measures in differentiating between LOPD and false positives and that Hex4 is an unreliable marker for this differentiation but aids in confirmation of IOPD. Regarding genotype, NBS2 helped to differentiate between unaffected infants and those with at least one c.-32-13T>G variant.

ERT was initiated for one patient in the cohort based on persistently elevated muscle biomarkers and decreasing scores on AIMS evaluation. ERT was initiated by 5 months of age with subsequent normalization of CK and notable improvement in AIMS percentile. Continued data collection for cases of LOPD identified by NBS will be important in helping to define indications for ERT initiation. Of note, there are no homozygotes to date for the common splice site variant in this cohort that has been started on ERT.

ERT for PD has been available since 2006. Patients with no functional enzyme are classified as Cross-Reactive Immunological Material (CRIM) negative. These individuals face the risk of developing antibodies to the enzyme when it is administered and recognized by the immune system as foreign. CRIM-positive patients may also develop antibodies, but they do not appear to interfere with treatment efficacy [3]. Prior to the use of immune modulation, CRIM-negative patients died or experienced major clinical decline while receiving ERT [3]. With the administration of immune modulation, however, CRIM-negative patients successfully receive ERT with consistently negative antibody titers or have a transient antibody response that diminishes over time [3].

Newborn screening for Pompe disease resulted in the unexpected identification of a cohort of newborns diagnosed with LOPD and suspected LOPD. The diagnosis of LOPD had historically been made following a diagnostic odyssey in children or adults with nonspecific muscular or respiratory complaints. We are now presented with the opportunity and challenge of managing LOPD before the onset of clinically apparent symptoms. Data collection on these patients is critical for developing management guidelines, particularly as they pertain to the initiation of ERT and improved classification of VUS.

## Figures and Tables

**Table 1:** Summary of genotype and phenotype data for the cohort. Biochemical markers presented were measured at the first evaluation at our center following the identification of a positive newborn screen.

Subj	Sex	Age at Initial Consult. (days)	NBS 1	NBS 2	Allele 1	Allele 2	GAA	Hex4	CK	AST	ALT	Initial EKG	Initial Echo	Diagnosis
1	M	28	1.07	0.38	c.-32-13T>G	c.456_457dupGA (p.Thr153Argfs*14)	1.1	8.1	510	158	39	Normal	PFO	Late Onset
2	M	14	1	0.4	c.-32-13T>G	c.2237G>A (p.Trp746X)	2.6	6.1	464	112	50	Right axis deviation	PFO	Late Onset
3	M	54	2.1	1.75	combined pseudodef alleles c.1726G>A(p.Gly576Ser)/c.2065G>A(p.Glu689Lys)	combined pseudodef alleles c.1726G>A(p.Gly576Ser)/c.2065G>A(p.Glu689Lys)	2.2	4.9	137	42	36	Normal	Normal	Pseudodeficiency
4	F	16	0.98	0.58	c.1124G>A (p.Arg376His)	c.2105G>T (p.Arg702Leu)	1.2	3	85	64	20	Normal	PFO	Late Onset
5	F	15	1.59	0.95	c.-32-13T>G	c.1888+5G>t	2.7	3.3	65	73	19	Normal	Normal	Unknown Onset
6	M	19	1.8	1.45	c.-32-13T>G		4.2	6.5	113	51	22	Normal	PFO	Carrier
7	F	17	1.28	0.63	c.-32-13T>G	c.2c.1935C>A (p.Asp645Glu)60C>T (p.Arg8c.1935C>A (p.Asp645Glu)4X)	3	4.5	421	125	50	Hypertrophy	Hypertrophy	Late Onset
8	F	15	0.7	0.34	c.2560C>T (p.Arg854X)	c.-32-13T>G	1.4	7.6	542	152	55	Normal	Normal LV size, but qualitatively subtle hypertrophy of LV apex, PFO	Late Onset
9	F	23	1.56	1.64	c.1210G>A (p.Asp404Asn)		3.4	4.5	107	105	18	Normal	Normal	Carrier
10	M	27	1.06	0.26	c.-32-13T>G	c.-32-13T>G	6.4	8.5	140	73	39	Normal	PFO	Late Onset
11	M	23	1.95	1.49	c.1655T>C (p.Leu552Pro)	c.266G>A (p.Arg89His)	2.1	3.8	88	56	36	Normal	Normal	Unknown Onset
12	M	32	0.56	0.62	c.-32-13T>G	c.-32-13T>G	3.8	4.8	122	78	42	Normal	PFO, PDA	Late Onset
13	F	21	1.02	1.22	c.2560C>T (p.Arg854X)	c.1888+5G>T	3.8	4.6	69	96	44		triscupid regurgitation, dilatation and hypertrophy of RV, normal LV, transverse arch and isthmus hypoplasia	Unknown Onset
14	F	25	1.77	0.84	c.-32-13T>G	c.-32-13T>G		12.6	155	71	60		Normal	Late Onset
15	F	30	1.83	1.74	c.258C>A (p.Pro86=)		3.7	5.6	55	55	30			Normal
16	F	23	2.02	1.07	c.1935C>A (p.Asp645Glu)	combined pseudodef alleles c.1726G>A(p.Gly576Ser)/c.2065G>A(p.Glu689Lys)	2.3	5	108	103	40			Carrier
17	F	22	2.02	1.46	c.1979G>A (p.R660H)		4.73	3.4	60	71	45			Carrier
18	F	24	1.97	1.27	c.-32-13T>G	combined pseudodef alleles c.1726G>A(p.Gly576Ser)/c.2065G>A(p.Glu689Lys)	1.8	3.3	86	80	41			Unknown Onset
19	F	29	1.12	0.62	c.2238G>C (p.W746C)	c.2238G>C (p.W746C)	2.5		48	73	28	Normal	cor triatriatum dexter	Late Onset
20	F	25	1.64	1.1	c.-32-13T>G	c.650C>T (p.P217L)		5.7	68	65	35	Normal	PFO	Unknown Onset
21	M	28	0.65	0.34	c.525delT (p.Glu176ArgfsTer45)	c.1694_1697delTCTC		30.2	846	189	91	Diffuse T-wave inversions, biventricular hypertrophy with strain	Hypertrophy	Infantile Onset
22	F	35	1.68	0.79	c.-32-13T>G	c.156_157delTC p.His53fs	2.4	7	467	166	73	Normal	PFO	Late Onset
23	M	17	1.03	0.77	c.-32-13T>G	c.-32-13T>G	1.8	5.2	275	150	42	Right axis deviation	Normal	Late Onset
24	M	37	1.94	2.06	c.1478C>T (p.P493L)	c.1194+3G>C	6.05	4.4	86				PFO, trivial left to right atrial shunt.	Unknown Onset
25	F	24	1.98	1.17	c.1552-3C>G	c.1378G>A (p.Glu460Lys)		3.7	123	91	46	Normal	PFO	Unknown Onset
26	M	36	1.5	1.13	c.752C>T(p.Ser261Leu)/c.761>T(p.Ser264Leu)	combined pseudodef alleles c.1726G>A(p.Gly576Ser)/c.2065G>A(p.Glu689Lys)	3.75	3.2	115	43	17			Carrier
27	M	55		1.99	combined pseudodef alleles c.1726G>A(p.Gly576Ser)/c.2065G>A(p.Glu689Lys)	combined pseudodef alleles c.1726G>A(p.Gly576Ser)/c.2065G>A(p.Glu689Lys)	12.4	2.4	126	64	48			Pseudodeficiency
28	M	37		0.42	c.-32-13T>G	c.1103G>A(p.Gly368Asp)		7.2	407	126	85	Concern for BVH on EKG	PFO	Unknown Onset
29	M	35	1.56	1.04	c.2560C>T (p.Arg854X)		2.55		111	74	35			Carrier
30	M	22	1.1	0.42	c.2238G>C	c.2065G>A (p.Glu689Lys)	2.4	3.3	108	32	29	Normal		Late Onset
31	F	52	1.59		c.-32-13T>G	c.-32-13T>G		2.8	173	80	47	Normal	PFO	Late Onset
32	F	25	0.86	0.58	c.2238G>C (p.Trp746Cys)	c.2238G>A (p.Trp746Ter)		0.6	117	74	55	Normal	Tiny PFO, mildly increased trabeculations at LV apex, LPA mildly hypoplastic	Late Onset
33	M	26	0.63	0.34	c.671G>A (p.Arg224Gln)	c.2467A>T (p.Ile823Phe)	1.44		120	40	21	Normal	Normal	Unknown Onset
34	F	33	0.58	1.84	c.1A>G (p.M1?)	combined pseudodef alleles c.1726G>A(p.Gly576Ser)/c.2065G>A(p.Glu689Lys)		8.4	241	44	14	Normal	Normal	Unknown Onset
35	F	98	1.71	1.56	c.2560C>T (p.Arg854X)		2.3							Carrier
36	M	25	0.84	0.49	c.1438-1G>C	c.-32-13T>G	2.1	6.6	641	173	62	Normal	Normal	Late Onset
37	M	NA		1.18	c.271G>A (p.Asp91Asn)									
38	M	21	1.1	1.08	c.1935C>A (p.Asp645Glu)	c.2065G>A (p.Glu689Lys)	3.2	3.3	69	93	32			Carrier
39	M	25	0.97	0.33	c.2238G>C (p.Trp746Cys)	c.2281deGinsAT	0.93	5.3	145	72	43	Normal	Normal	Late Onset
40	F	40	1.85	1.8	combined pseudodef alleles c.1726G>A(p.Gly576Ser)/c.2065G>A(p.Glu689Lys)	combined pseudodef alleles c.1726G>A(p.Gly576Ser)/c.2065G>A(p.Glu689Lys)								Pseudodeficiency
41	M	16	8.3		c.-32-13T>G	c.-32-13T>G		4.1	103	150	46	Normal	Normal	Late Onset
42	M	28	1.19	1.27	c.1655T>C(p.Leu552Pro)	c.1888+5G>T	0.43	3.9	110	60	25	Borderline QTc	Normal	Late Onset
43	F	27	1.75	1.5	c.752C>T; 761 C>T p.(Ser251Leu; Ser425Leu)	combined pseudodef alleles c.1726G>A(p.Gly576Ser)/c.2065G>A(p.Glu689Lys)		3.8	68	83	18			Carrier
44	M	48	0.88	0.77	c.-32-13T>G	c.-32-13T>G	3.5		204	63	34			Late Onset
45	F	22		0.65	c.-32-13T>G	c.-32-13T>G			528	109	60			Late Onset

## Data Availability

The original contributions presented in this study are included in the article. Further inquiries can be directed to the corresponding author.
